# Theoretically quantifying the direct and indirect benefits of vaccination against SARS-CoV-2 in terms of avoided deaths

**DOI:** 10.1038/s41598-022-12591-w

**Published:** 2022-05-25

**Authors:** Greg Scutt, Mike Cross, David Waxman

**Affiliations:** 1 Medicines Optimisation Research Group, School of Applied Sciences, University of Brighton, Brighton, UK; 2 Pharmacy Department, University Hospitals Sussex NHS Foundation Trust, Brighton, UK; 3 Centre for Computational Systems Biology, ISTBI, Fudan University, Shanghai, People’s Republic of China

**Keywords:** Medical research, Epidemiology

## Abstract

The Coronavirus Disease 2019 (COVID)-19 pandemic has placed unprecedented pressures on societies around the world. Successful vaccines, developed against the spike protein of the Severe Acute Respiratory Syndrome Virus 2 (SARS-CoV-2) virus, offer hope that new hospitalisations and new deaths will subside. However, vaccination takes place in a dynamic environment. For example, new variants of the disease may occur where the effectiveness of a vaccine lies below that of the original target of the vaccine, while changes in the behaviour of a population are accompanied by a changed basic reproduction number. Here, we aim to understand how changes in values of basic parameters affect the benefits of vaccination at the *direct level*, of the individuals vaccinated, and at the *indirect level*, of the wider, unvaccinated community. We work within the framework of a Susceptible-Infected-Recovered model, and produce a metric for the benefits of vaccination, at both direct and indirect levels, in terms of the number of avoided deaths. Taking into account the initial prevalence of a SARS-CoV-2 infection, the mortality rate of the disease, the basic reproduction number, the vaccination rate, and the effectiveness of a vaccine, we explore how these basic parameters affect the benefits of vaccination. We find a range of situations where indirect benefits of vaccination outweigh direct benefits. This especially occurs at lower rates of vaccination (20% – $40\%$) and intermediate values of the basic reproduction number (1–1.5). The indirect benefits can be substantial, in some cases being more than 400% of the direct benefits. For an initial prevalence of SARS-CoV-2 infection of 2%, a basic reproduction number of 1.2, a mortality rate of 2%, and a vaccine effectiveness of 95%, our findings show, for a population of 500,000 people, where 100,000 susceptible individuals are vaccinated, that approximately 2200 deaths are avoided. However, approximately 600 of these deaths are avoided amongst vaccinated individuals, while approximately 1600 deaths are avoided in the wider, unvaccinated community.

## Introduction

The recent Coronavirus Disease-2019 (COVID-19) pandemic has placed unprecedented health, social, and economic pressures on different societies around the world^1^. The development and licensing of vaccines against components of the Severe Acute Respiratory Syndrome Coronavirus 2 (SARS-CoV-2) virus has provided hope that hospitalisations and deaths from the disease can be suppressed, and that societies can begin to rebuild^2,3^.

The effectiveness of local and national vaccination programmes in reducing hospitalisations, deaths, and viral transmission, is partly dependent upon adherence by the public to vaccination^4^. Indeed some patients, who may not mount a full, and sustained immune response to vaccination (e.g., the immunocompromised^5^) rely on population-level immunity (so called *herd-immunity*) to obtain protection. Several other phenomena, which change transmission rates of the disease, also play a central role in the effectiveness of a vaccination program. These phenomena include:

(i) changes in behaviour of the population, which can reduce or increase the reproduction number, with commensurate effects on transmission^6,7^;

(ii) a reduction in the number of susceptible individuals over time, which manifests itself in a decreasing effective reproduction number^8^;

(iii) increases in the vaccination rate, which makes it less likely that infected individuals come into contact with susceptible individuals^9^.

(iv) the emergence of new viral strains, or variants that demonstrate vaccine escape

However, the complex interplay of such phenomena takes place in a dynamic situation, where vaccines change their performance, because of changes in the behaviour of the population, and alterations of the disease’s structure^9^.

Any response to a pandemic, particularly in a changing situation, requires a clear understanding of the factors affecting the benefits of a vaccination programme. At the population-wide level, this appears to be difficult to state because vaccination has different benefits to different groups within the population. Individuals who are vaccinated acquire a level of protection against infection and death from the disease, and hence gain a *direct benefit* of vaccination. The wider unvaccinated community suffers a reduction in deaths, due to the presence of vaccinated individuals, who modify transmission of the disease. These gains are termed the *indirect benefits* of vaccination (and include so-called *herd immunity*^10^). To date, there is evidence which suggests that vaccination can reduce viral transmission and onward infection to early variants of SARS-CoV-2^11^. However, for more recent variants, *household* transmission from vaccinated index cases is similar from those who are unvaccinated^12^. Nevertheless, viral load appears to decline at a faster rate in individuals who are vaccinated, suggesting that the window of transmissability may be reduced by SARS-Cov-2 vaccination^12^. We can therefore ask about which of the two groups, the vaccinated and the unvaccinated, benefits most from vaccination, and here we provide the means to quantify the different benefits. We shall adopt the simple metric of the *reduction in the number of deaths* of the two groups due to vaccination, but shall often frame matters in the language of *avoided deaths*, where vaccination avoids deaths that *would* have occurred in the *absence* of vaccination.

Quantifying the benefits of vaccination in terms of avoided deaths might be an important way of presenting the issue of vaccination for the public, especially in times where vaccination take up is incomplete. This metric may also be of importance to policy makers who, by understanding the determinants of the number avoided deaths, at the direct and indirect levels, will be in a position to make informed decisions about where best to put resources during a spike in infections: acute care, vaccination, or a combination of these. Importantly, this way of quantifying benefits may also provide information about the most appropriate time to relax social and economic restrictions, given a rising vaccination rate.

In summary, in this work we provide estimates of avoided deaths, based on a variant of a Susceptible-Infected-Recovered (SIR) model, and proceed by outlining a system of equations for this model. This allows numerical estimation of the reduction in deaths that occur when some members a population are vaccinated. We investigate the implications of these equations for a local population where SARS-CoV-2 is actively transmitting, when a one-off vaccination event has been implemented. We go on to explore the relationship between the numbers of avoided deaths at the direct and indirect levels, for different vaccination rates, different values of the basic reproduction number, and different levels of effectiveness of the vaccine. Numerical results, based on the SIR model, are supplemented by analytical approximations that expose the essential parameter dependencies of predictions of the model.

## Methods

In this work we adopt a variant of a discrete time SIR model (cf.^13^) where we incorporate vaccination that fully protects against dying of the disease, but is not $100\%$ effective against infection, with some vaccinated individuals remaining susceptible to the disease.

All results presented in the main text are derived and explained in greater detail in the  Supplementary Information.

### Assumptions

The following list contains the key assumptions made in this work, and also serves to establish the notation adopted.

- The mean infectious period for an individual, referred to as the *serial interval*, is denoted by $\tau$. We work in discrete time, which is time measured in units of the serial interval, $\tau$. The discrete time adopted is labelled by *n*, which takes the values $0,1,2,\ldots$. At discrete time *n*, the actual time is $n\times \tau$. In what follows, when we refer to time, we shall mean the *discrete time*, *n*, and the *initial time* refers to $n=0$.
- The *basic reproduction number* is denoted by $\mathbb {R}$. This is a constant whose value corresponds to the number of new infections that are produced by each infected individual, within the serial interval, when infections within the population are rare, and essentially all individuals are susceptible to the disease. As we shall see shortly, the basic reproduction number, $\mathbb {R}$, is a parameter whose value influences the entire time-course of an infection, not just when infections are rare. In the present work the basic reproduction number, $\mathbb {R}$, plays the role of a *phenomenological parameter*, whose value can be established by observations and experiments. In a more microscopic theory, where fundamental processes are directly incorporated into the model, the reproduction number then emerges as a composite parameter, that is built out of the parameters describing the fundamental processes (see, for example, Tiomela et al.^14^).
- The population, at any time, consists of three categories of individual, described as *susceptible*, *infected* and *recovered*. Susceptible individuals are those who have not been infected but can become infected in the future. Infected individuals have the disease during the serial interval, and during this time can transmit the disease to susceptible individuals. At the end of the serial interval, infected individuals either die or become recovered individuals who can no longer become infected, thus no infected individuals remain in the infected category after one time step. At discrete time *n* the numbers of individuals in the three categories are denoted by $S_{n}$, $I_{n}$ and $R_{n}$, respectively.
- At the initial time ($n=0$), the the fraction of the population that is infected, *p*, is termed the *prevalence* of the disease ($0\le p\le 1$).
- At the initial time ($n=0$), vaccination is carried out on some susceptible individuals in the population. The fraction of all susceptible individuals that are vaccinated, *v*, is termed the *vaccination rate* ($0\le \nu\le 1$).
- The probability that a vaccinated individual is not susceptible to the disease is denoted by $\varepsilon$ and termed the *effectiveness* of the vaccine ($0\le \varepsilon \le 1$). The probability that a vaccinated individual remains susceptible to the disease is $1-\varepsilon$. All vaccinated individuals that remain susceptible are, as far as contracting and transmitting the disease are concerned, assumed to be indistinguishable from unvaccinated individuals.
- All infected individuals, who have not previously been vaccinated, are assumed to have a probability of *m* of dying of the disease, by the end of the serial interval ($0\le m\le 1$). We term *m* the *mortality rate*. Note that we do not incorporate births, or deaths from causes other than the disease (*other deaths*), into the model. We assume the effects we wish to capture in this work (direct and indirect effects of vaccination) reveal themselves over a relatively short period of time, where the outcomes, on the population, of births and other deaths, can be neglected. If, however, births and other deaths have the combined effect of causing significant changes in population number over time-intervals of relevance, then their effects need to be incorporated into the model (see Eichneret. al. and Samera et al.^15,16^).
- We assume a spatially homogeneous population, i.e., one that is well-mixed.
- We treat the dynamics of the population *deterministically*, by setting the number of individuals in the three categories to their expected values, and hence ignore statistical fluctuations.

### Dynamics

In the variant of the discrete time SIR dynamics adopted in this work, vaccination of susceptible individuals occurs at the initial time ($n=0$). Immediately before vaccination there are $Q=(1-p)N$ susceptible individuals and *pN* infected individuals. Immediately after vaccination, there are $(1-\nu)Q$ unvaccinated individuals, and *vQ* vaccinated individuals. Of the vaccinated individuals, $\varepsilon \nu Q$ are *not* susceptible to the disease and are immediately promoted to the recovered category, while $(1-\varepsilon )\nu Q$ vaccinated individuals remain susceptible. The susceptible category thus contains $(1-\nu)Q$ unvaccinated individuals and the $(1-\varepsilon )\nu Q$ vaccinated individuals who remain susceptible. It follows that the initial numbers of susceptible, infected and recovered individuals are $S_{0}=(1-\varepsilon \nu)Q$, $I_{0}=pN$ and $R_{0}=\varepsilon \nu Q$, respectively. These initial values are combined with a system of equations that describes the dynamics of the problem, where some susceptible individuals become infected individuals, while at the end of a serial interval, existing infected individuals may die (if unvaccinated) or become recovered individuals, hence no infected individual remains infected after one time-step.

The dynamics involves an effective mortality rate, $m_{n}$, defined by

1 $$\begin{aligned} m_{n}=\left\{ \begin{array}{lll} m &{} \text {if }n=0, &{} \\ &{} &{} \\ f\times m &{} \text {if }n>0, &{} \end{array} \right. \quad \quad \text {where}\quad \quad \quad f=\dfrac{1-\nu}{1-\varepsilon \nu} \end{aligned}$$

which is required since the composition of infected individuals differs at different times. At the initial time ($n=0$) all infected individuals are unvaccinated and hence have mortality rate *m*. For all positive times ($n>0$) only a fraction *f* of infected individuals arise from unvaccinated individuals, and these individuals are subject to mortality at rate *m*; the remainder of infected individuals arise from vaccinated individuals and are not subject to mortality from the disease.

The system of equations for the SIR model is given by

2 $$\begin{aligned} S_{n+1}&=S_{n}-\mathbb {R}S_{n}I_{n}/N\nonumber \\&\nonumber \\ I_{n+1}&=\mathbb {R}S_{n}I_{n}/N\\&\nonumber \\ R_{n+1}&=R_{n}+I_{n}-m_{n}I_{n}\nonumber \end{aligned}$$

where the basic reproduction number, $\mathbb {R}$, determines how the meeting of susceptible and infected individuals leads to new infections.

We note that the quantity $S_{n}+I_{n}+R_{n}$ is the size of the population at time *n*. In the absence of mortality ($m=0$) the size of the population is constant, but for non-zero mortality, the size decreases over time. This decrease is at a very low rate if $mfI_{n}/N\ll 1$ as is found for the parameter ranges considered in this work.

The form of the number of new infections produced at time $n+1$, namely $I_{n+1}=\mathbb {R}S_{n}I_{n}/N$, has the property that when the number of infections is low ($I_{n}\ll N$), and the number of susceptible individuals is close to the initial population size ($S_{n}\simeq N$), we obtain $I_{n+1}\simeq \mathbb {R}I_{n}$. That is, in this regime, each infected individual produces approximately $\mathbb {R}$ new infections.

More generally, disease transmission is influenced by the value of the basic reproduction number, $\mathbb {R}$, irrespective of whether infections are rare or common. Disease transmission can be naturally described by an *effective reproduction number*, written $\mathbb {R}_{n}$, that depends on the number of susceptible individuals, and the time, and is given by $\mathbb {R}_{n}=\mathbb {R}S_{n}/N$. In terms of $\mathbb {R}_{n}$ the second line of Eq. (2) can be written as $I_{n+1}=\mathbb {R}_{n}I_{n}$, indicating that at time *n* each infected individual infects $\mathbb {R}_{n}$ susceptible individuals. The critical value of $\mathbb {R}_{n}$ in this model is unity, which occurs at $S_{n}=N/\mathbb {R}$, and signals the onset of herd immunity. That is, when $\mathbb {R}_{n}<1$ the number of infected individuals decreases at each time step.

A mathematical property of the SIR model adopted in this work is that the quantity $I_{n}/N$ (the number of infected individuals, scaled by the initial population size, *N*) is independent of *N*. The results we present below are associated with $I_{n}/N$, and hence apply for any *N*.

The number of infected individuals at time *n*, namely $I_{n}$, depends on the parameters $\mathbb {R}$, *p*, $\varepsilon$, *v* and *N*. In what follows, we only indicate the dependence of $I_{n}$ on those parameters needed to clarify the exposition, for example writing $I_{n}(\nu)$ instead of exhibiting dependence on all five parameters. We will similarly suppress nonessential parameter dependencies of other quantities.

## Results

We shall derive expressions for different *benefits* of vaccination (direct, indirect and total), in terms of the number of infected individuals, $I_{n}$. Additionally, using an approximation for $I_{n}$, we shall also provide approximate results for the different benefits of vaccination, which will aid our understanding, by exposing essential dependencies on the parameters of the model.

We will make repeated use of the approximation for $I_{n}$, and sketch here the basis of this approximation (see the  Supplementary Information for full details). We assume that: (1) $S_{n}$ and $I_{n}$ change sufficiently slowly with *n* that they can be treated as continuous functions of *n*; (2) differences, such as $I_{n+1}-I_{n}$ can be replaced by a derivative with respect to *n*, i.e., $I_{n+1}-I_{n}\simeq dI_{n}/dn$; (3) the quantity $\ln \left( 1-\mathbb {R}I_{n}/N\right)$ can be approximated by $-\mathbb {R}I_{n}/N$. These assumptions, applied in the SIS system of equations (Eq. 2), lead to

3 $$\begin{aligned} I_{n}\simeq \frac{N\alpha ^{2}}{2\mathbb {R}}{\text {sech}}^{2}\left( \frac{\alpha n}{2}+\frac{\beta }{2}\right) \end{aligned}$$

where $\alpha$, $\beta$ and some other parameter combinations that arise below, are given by

4 $$\begin{aligned}{}\begin{array}{lllll} L=\ln [\mathbb {R}(1-\varepsilon \nu)(1-p)], &{} &{} \alpha =\sqrt{2\mathbb {R} p+L^{2}}, &{} &{} \beta =\ln \left( \dfrac{\sqrt{2\mathbb {R}p+L^{2}}-L}{\sqrt{2\mathbb {R}p+L^{2}}+L}\right) ,\\ &{} &{} &{} &{} \\ L_{0}=\ln [\mathbb {R}(1-p)], &{} &{} \alpha _{0}=\sqrt{2\mathbb {R}p+L_{0}^{2}}. &{} &{} \end{array} \end{aligned}$$

### Benefits of vaccination

To determine the direct and indirect benefits of vaccination, we make a comparison of two populations. One population, as described above, is where vaccination has been carried out in a proportion *v* of all susceptible individuals. In the other population, no vaccinations have been carried out, but in all other regards, the two populations are closely comparable (all parameter values except *v* are identical).

In the population subject to vaccination, there are *Q* susceptible individuals immediately before vaccination. Immediately after vaccination there are *vQ* vaccinated individuals. The direct benefit of vaccination is measured by tracking the fate of *vQ* of the *Q* susceptible individuals in the *unvaccinated* population. The deaths that ultimately occur to the *vQ* tracked individuals are deaths that do not occur in the corresponding vaccinated population (vaccinated individuals are protected from dying of the disease). The deaths that occur are thus *avoided* deaths of the vaccinated individuals, and represent the *direct benefit* of vaccination.

We find vaccinating *vQ* susceptible individuals has the benefit of directly avoiding $\nu m\sum _{n=1}^{\infty }I_{n}(0)$ deaths, where $I_{n}(\nu)$ is the number of infected individuals in a population with vaccination rate *v*.

The measure of the direct benefit of vaccination we shall adopt is the number of directly avoided deaths, scaled by both the mortality rate, *m*, and the initial population size, *N*. That is, we define

5 $$\begin{aligned} \Delta _{D}&= \begin{array}{l} \text {measure of the direct}\\ \text {benefit of vaccination} \end{array} =\frac{\text {total number of directly avoided deaths}}{\text {mortality rate}\times \text {initial population size}}\nonumber \\&\nonumber \\&=\frac{\nu m\sum _{n=1}^{\infty }I_{n}(0)}{m\times N}=\frac{\nu\sum _{n=1}^{\infty }I_{n}(0)}{N}\nonumber \\&\nonumber \\&\simeq \nu\frac{\alpha _{0}+L_{0}}{\mathbb {R}} \end{aligned}$$

where the final line is an approximation that follows from Eq. (3). We shall often refer to $\Delta _{D}$ simply as the *direct benefit*. It depends on the parameters $\mathbb {R}$, *p* and *v* but is independent of *N*, *m* and $\varepsilon$, hence $\Delta _{D} \equiv \Delta _{D}\left( \mathbb {R},p,\nu\right)$.

From Eq. (5), the *v* dependence of $\Delta _{D}$ is particularly simple. The $I_{n}(0)$ are properties of an unvaccinated population and hence have no dependence on *v* or $\varepsilon$. Thus $\Delta _{D}$ is proportional to *v* and independent of $\varepsilon$, and the approximate expression in Eq. (5) gives an indication of the dependence on $\mathbb {R}$ and *p*. In an infected population, described by the parameters $\mathbb {R}$, *p*, *N* and *m*, vaccinating a proportion *v* of the susceptible individuals has the direct effect of avoiding $N\times m\times \Delta _{D}\left( \mathbb {R} ,p,\nu\right)$ deaths of vaccinated individuals.

In a similar but more involved way we can calculate the indirect effect of vaccination. In the population where vaccination is carried out, the number of susceptible individuals that are *not* vaccinated is $(1-\nu)Q$. The indirect benefit of vaccination is measured by tracking the fate of this number of individuals in the susceptible category of an unvaccinated population. The difference in numbers of deaths, because of the *presence* of vaccinated individuals, constitutes the *indirect benefit* of vaccination.

We find there are $(1-\nu)m\sum _{n=1}^{\infty }I_{n}(0)-fm\sum _{n=1}^{\infty }I_{n}(\nu)$ fewer (or avoided) deaths of unvaccinated individuals in the population where some individuals are vaccinated.

The measure of the indirect benefit of vaccination we shall adopt is the number of indirectly avoided deaths, scaled by both the mortality rate, *m*, and the initial population size, *N*. That is, we define

6 $$\begin{aligned} \Delta _{I}&= \begin{array}{l} \text {measure of the indirect}\\ \text {benefit of vaccination} \end{array} =\frac{\text {total number of indirectly avoided deaths}}{\text {mortality rate}\times \text {initial population size}}\nonumber \\&\nonumber \\&=\frac{(1-\nu)m\sum _{n=1}^{\infty }I_{n}(0)-fm\sum _{n=1}^{\infty }I_{n} (\nu)}{m\times N}\nonumber \\&\nonumber \\&\simeq (1-\nu)\frac{\alpha _{0}+L_{0}}{\mathbb {R}}-f\frac{\alpha +L}{\mathbb {R} }. \end{aligned}$$

where the final line is an approximation that follows from Eq. (3). We shall refer to $\Delta _{I}$ simply as the *indirect benefit*. It depends on the parameters $\mathbb {R}$, *p*, $\varepsilon$ and *v*, but is independent of *N* and *m*, hence $\Delta _{I}\equiv \Delta _{I}\left( \mathbb {R},p,\varepsilon ,\nu\right)$ and the approximate expression in Eq. (6) gives an indication of the dependence on these parameters. In an infected population described by the parameters $\mathbb {R}$, *p*, *N* and *m*, vaccinating a proportion *v* of susceptible individuals will have the indirect effect of avoiding $N\times m\times \Delta _{I}\left( \mathbb {R},p,\varepsilon ,\nu\right)$ deaths of unvaccinated individuals.

The *total benefit* of vaccination is the sum of direct and indirect benefits. We write the total benefit as $\Delta _{T}$. Thus

7 $$\begin{aligned} \Delta _{T}&= \begin{array}{l} \text {measure of the total}\\ \text {benefit of vaccination} \end{array} =\frac{\text {total number of avoided deaths}}{\text {mortality rate} \times \text {initial population size}}=\Delta _{D}+\Delta _{I}\nonumber \\&\nonumber \\&=\left[ \sum _{n=1}^{\infty }I_{n}(0)-f\sum _{n=1}^{\infty }I_{n}(\nu)\right] /N. \end{aligned}$$

This has the approximation $\Delta _{T}\simeq \left( \alpha _{0}+L_{0}\right) /\mathbb {R}-f\left( \alpha +L\right) /\mathbb {R}$.

**Figure 1:**
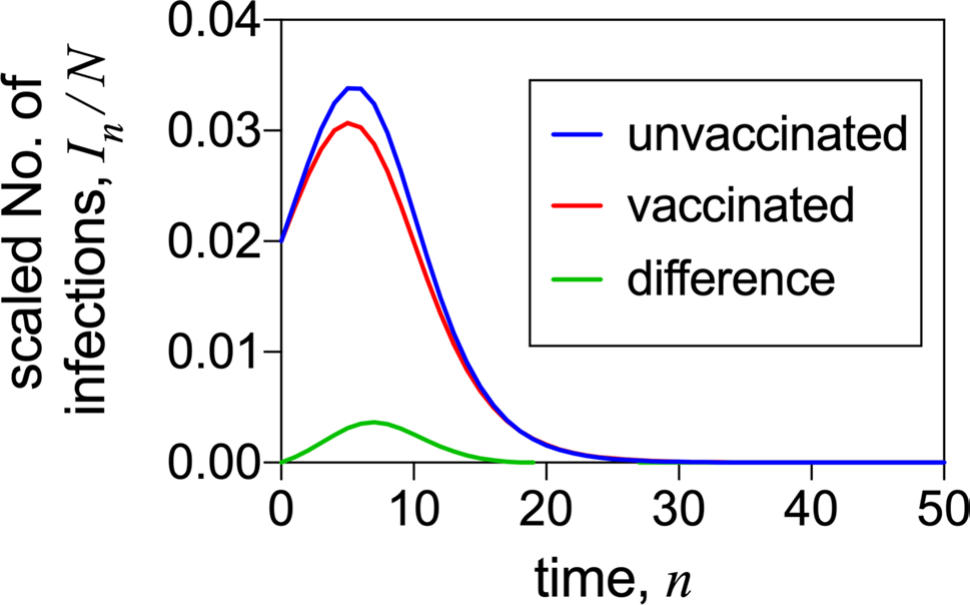
Number of infected individuals. We plot infection numbers, as a function of the discrete time, *n*, in the presence and absence of vaccination. The ratio plotted, $I_{n}/N$, is independent of both the mortality rate, *m*, and the initial population size, *N*, hence the figure applies for any values of *m* and *N*. The peak value of the number of infections is proportional to both the maximum number of individuals hospitalised and also the maximum number of individuals that die at any time. The parameter values adopted for the figure are: vaccination rate $\nu=0.02$, initial prevalence of the disease $p=0.02$, and basic reproduction number $\mathbb {R}=1.2$.

We note that the discrete time model, used in this work, can be numerically solved by direct iteration of the governing set of equations (Eq. 2), without the need of any dedicated methods, such as numerical differential equation solvers. A direct approach would be to specify the initial values, $S_{0}$, $I_{0}$ and $R_{0}$, and then use the set of equations in Eq. (2), with *n* set to 0, to determine $S_{1}$, $I_{1}$ and $R_{1}$. We would then use the corresponding equations in Eq. (2), with *n* set to 1, along with the (now) known values of $S_{1}$, $I_{1}$ and $R_{1}$, to determine $S_{2}$, $I_{2}$ and $R_{2}$, and so on. (As pointed out above, and in the Supplementary Information, it is actually advantageous to determine the scaled quantities $S_{n}/N$, $I_{n}/N$ and $R_{n}/N$, where *N* is the initial population size, rather than $S_{n}$, $I_{n}$ and $R_{n}$ themselves. The scaled quantities have the advantage of being independent of the initial population size, *N*.) From knowledge of $S_{n}$, $I_{n}$ and $R_{n}$ for a range of values of *n*, starting with $n=0$, we can determine all quantities of interest. In particular, using the set of equations in Eq. (2), we can illustrate the effect of vaccination on the number of infected individuals over time. In Fig. 1, we plot the number of infected individuals in the absence and presence of vaccination ($I_{n}(0)$ and $I_{n}(\nu)$, respectively), as functions of the time, *n*, along with their difference, $I_{n} (0)-I_{n}(\nu)$. This Figure illustrates how vaccination decreases the numbers of cases of infection over time, and this decrease becomes converted to a decrease in the total number of deaths due to vaccination.

We next consider behaviour of the different benefits of vaccination, for parameter values relevant to SARS-CoV-2. We use an initial prevalence of $p=0.02$ and only consider this value of the parameter in what follows, where we explore effects of different values of the vaccination rate, *v*, the basic reproduction number, $\mathbb {R}$, and the vaccine effectiveness, $\varepsilon$.

For given values of the three parameters *v*, $\mathbb {R}$, and $\varepsilon$, we use a scaled version of Eq. (2) to determine the direct, indirect and total benefits of vaccination, $\Delta _{D}$, $\Delta _{I}$ and $\Delta _{T}$, respectively. As defined, these three benefits are independent of the mortality rate, *m*, and the initial population size, *N*, hence the figures of $\Delta _{D}$, $\Delta _{I}$ and $\Delta _{T}$ that follow apply for all values of *m* and *N*.

**Figure 2:**
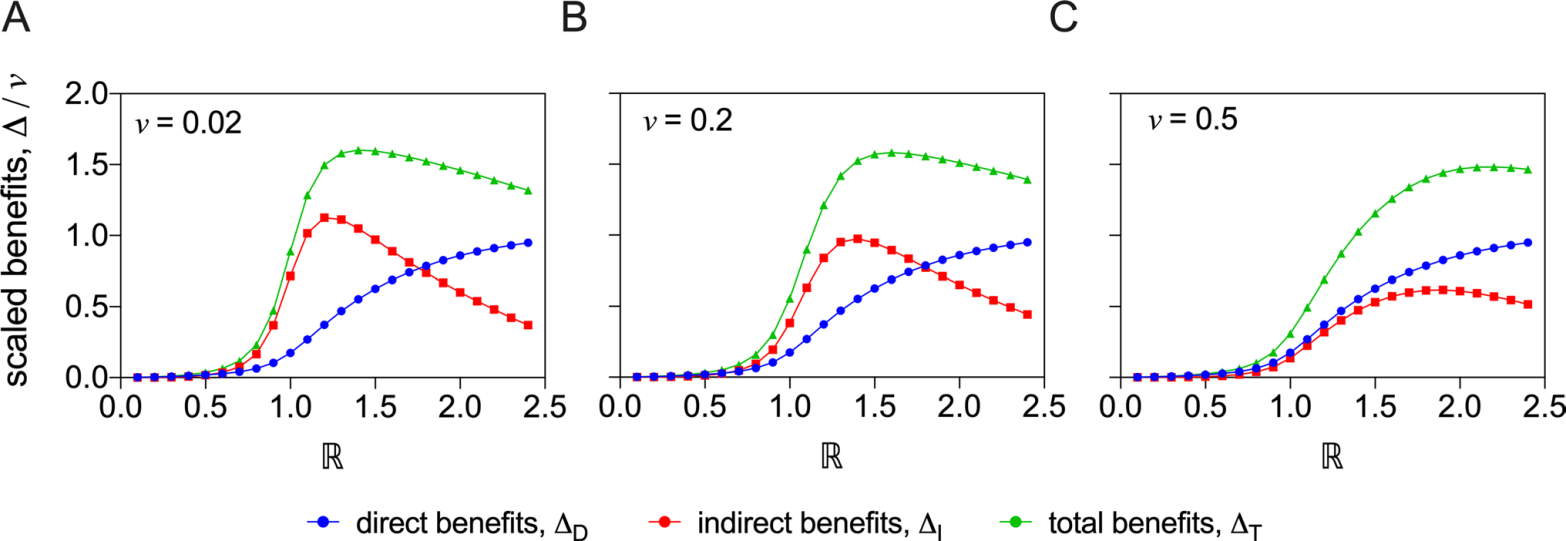
Benefits of vaccination. The direct, indirect and total benefits are plotted as functions of the basic reproduction number, $\mathbb {R}$. The effectiveness of the vaccine has been set to $\varepsilon =1.0$, and the vaccination rate, *v*, has been set to the values 0.02, 0.2 and 0.5 in Panels (**A**–**C**), respectively.

In Fig. 2, we plot $\Delta _{D}$, $\Delta _{I}$ and $\Delta _{T}$ as functions of $\mathbb {R}$, for three fixed values of *v* ($\nu=0.02$, $\nu=0.2$ and $\nu=0.5$; Panel A, B and C, respectively). In all three panels of the figure, the *direct benefit* of vaccination increases steadily as $\mathbb {R}$ increases and then appears to converge to the vaccination rate *v*. In accordance with calculation, the shape of the curve does not exhibit any dependence on the vaccination rate. Unlike the direct benefit, the *indirect benefit*, in all three panels, does not increase monotonically with $\mathbb {R}$. Rather, it exhibits initial increase, followed by a maximum and then decline at larger values of $\mathbb {R}$. At larger choices of *v* the location of the maximum is located at larger values of $\mathbb {R}$. The *total benefit* is a sum of direct and indirect benefits, and has behaviour that is less extreme but similar to that of the indirect benefit.

**Figure 3:**
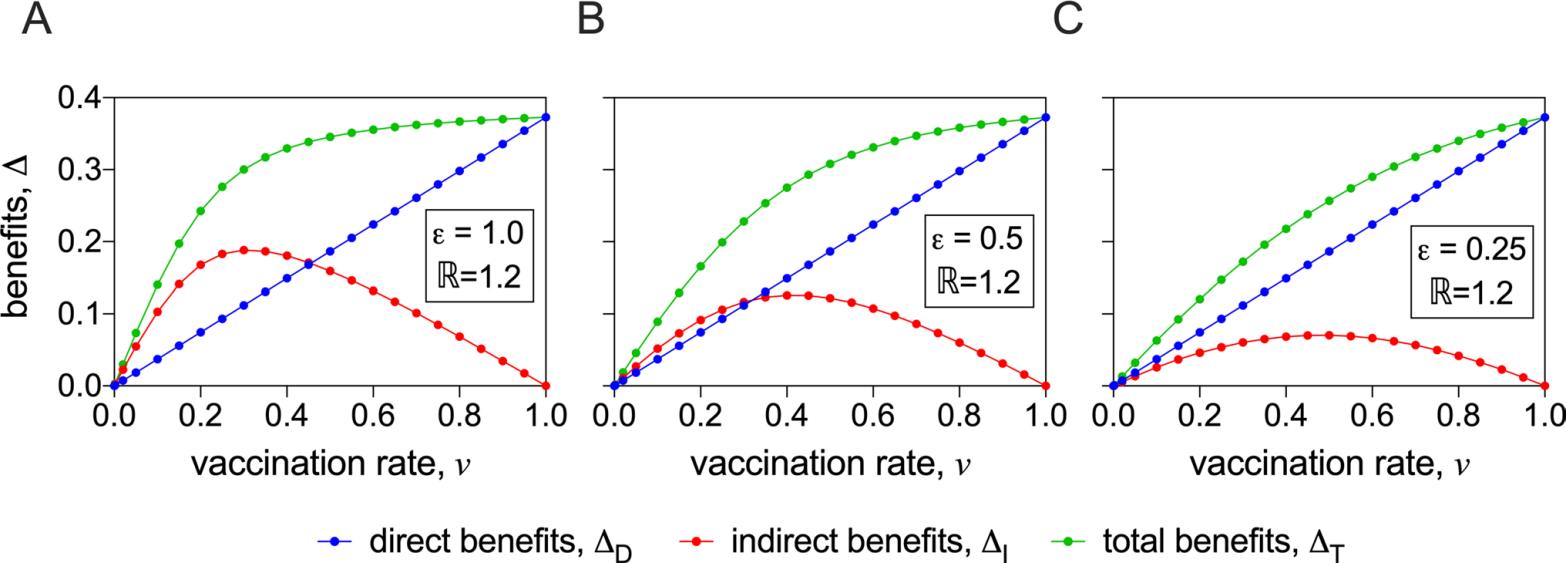
Benefits of vaccination. The direct, indirect and total benefits are plotted as functions of the vaccination rate *v*. The basic reproduction number has been set to $\mathbb {R}=1.2$, but the vaccine effectiveness, $\varepsilon$, has been set to the values 1.0, 0.5, and 0.25 in Panels (**A**–**C**), respectively.

In Fig. 3, the direct, indirect and total benefits are plotted as functions of *v*. To explore implications of different levels of effectiveness of the vaccine, $\varepsilon$, on the benefits of vaccination, the panels contain plots where $\varepsilon$ has been set to different values (1.0, 0.5, and 0.25 in Panels A, B and C, respectively). We observe the linear relationship between direct benefit and *v*, but the curves for indirect benefit exhibit a maximum at an intermediate value of *v*. For *highly effective* vaccines (Panel A, Fig. 3), the maximum occurs at a low vaccination rate, and indicates lower deaths, i.e., greater protection, in unvaccinated individuals, compared with vaccinated individuals. As $\varepsilon$ is decreased, the indirect benefit reduces until it lies below the direct benefit.

At a basic level, the indirect benefit *must* behave non-monotonically, as a function of *v*. When $\nu=0$ there are no benefits of any kind of vaccination, including no indirect benefit. Furthermore, when $\nu=1$ the only benefit of vaccination is direct, so again the indirect benefit is zero. Hence any non-zero value of the indirect benefit, at intermediate values of *v*, will exhibit non-monotonic behaviour, and as the figures show, this is in the form of a maximum.

**Figure 4:**
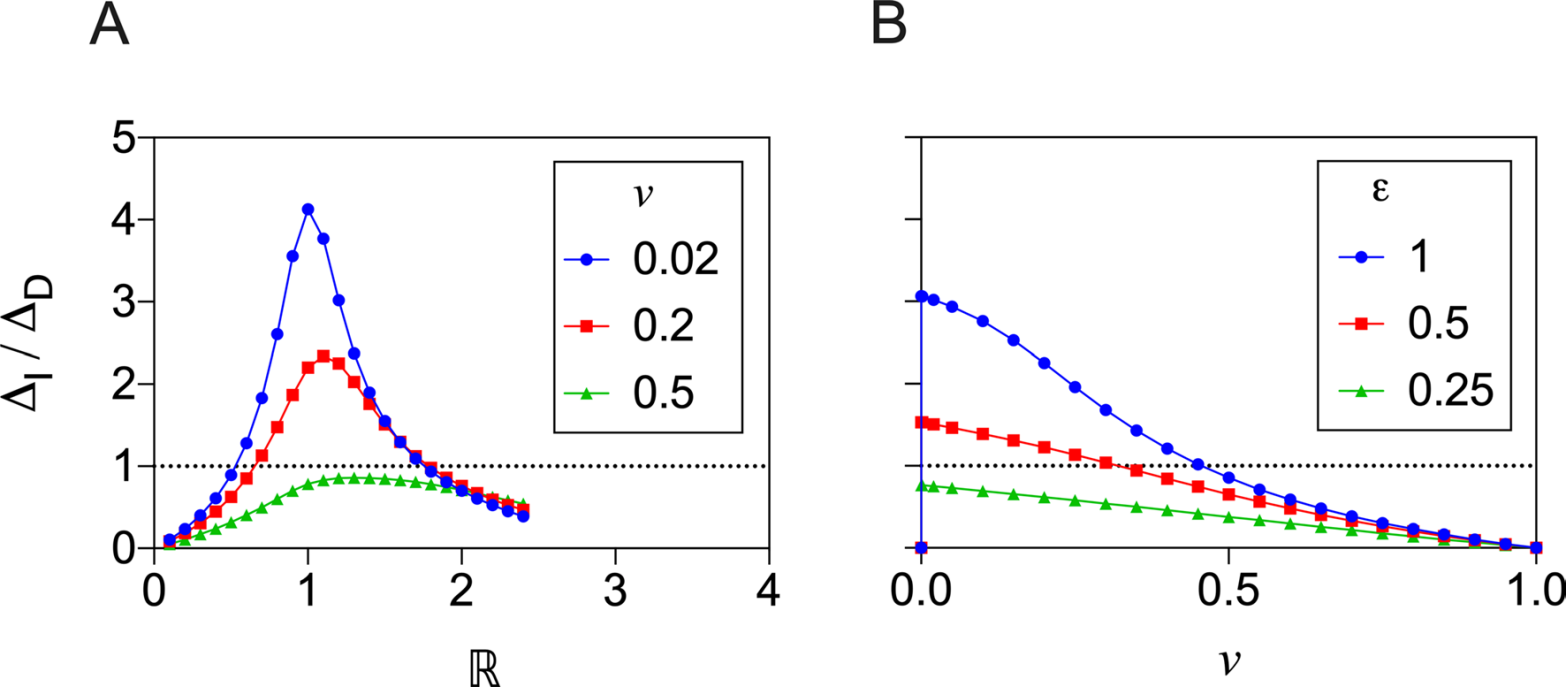
Ratio of indirect to direct benefits. The ratio $\Delta _{I} /\Delta _{D}$ is plotted as a function of the basic reproduction number, $\mathbb {R}$, (Panel **A**), and as a function of the vaccination rate, *v* (Panel **B**). In Panel (**A**), the ratio is plotted using data from Fig. 2 when the vaccine effectiveness, $\varepsilon$, is set to the value $\varepsilon =1.0$, while three different values of the vaccination rate, *v*, are used: 0.02, 0.2 and 0.5. In Panel (**B**), the ratio is plotted using data from Fig. 3 when the basic reproduction number, $\mathbb {R}$, is set to $\mathbb {R}=1.2$, while three different values if the vaccine effectiveness, $\varepsilon$, are used: 1.0, 0.5 and 0.25.

A prominent feature of Figs. 2 and 3 is that in some parameter regions the indirect benefit of vaccination exceeds the direct benefit. Generally, the ratio of indirect to direct benefits, $\Delta _{I}/\Delta _{D}$, depends on the parameter values $\mathbb {R}$, $\varepsilon$ and *v*. In Fig. 4, the ratio $\Delta _{I} /\Delta _{D}$ is plotted as a function of $\mathbb {R}$ (Panel A) and as a function of *v* (Panel B).

For Panel A of Fig. 4, the vaccine effectiveness, $\varepsilon$, is set to the value $\varepsilon =1.0$ while three different values of the vaccination rate, *v*, are used. The ratio $\Delta _{I}/\Delta _{D}$, plotted as as a function of the basic reproduction number, $\mathbb {R}$, can exceed unity, corresponding to the domination of direct benefits by indirect benefits. For the lowest vaccination rate considered, $\nu=0.02$, the ratio exceeds 4, and for the higher vaccination rate of $\nu=0.2$ the ratio exceeds 2. We note that when the ratio exceeds unity, it does so for an *intermediate range* of $\mathbb {R}$ values, not small $\mathbb {R}$ and not large $\mathbb {R}$.

For Panel B of Fig. 4, the basic reproduction number, $\mathbb {R}$, is set to the value $\mathbb {R}=1.2$ while three different values of vaccine effectiveness, $\varepsilon$, are used. The ratio $\Delta _{I}/\Delta _{D}$, plotted as as a function of *v*, can again exceed unity, however when it does, this occurs at smaller values of *v*. The largest values of the ratio occur at the larger values of $\varepsilon$.

## Discussion

Behavioural change, and more recently vaccination, have been the primary tools used by different nations to reduce infections, hospitalisations and, ultimately, deaths due to the SARS-CoV-2 virus^17^. The effectiveness of these interventions are nonetheless subject to change. For example, new variants of a virus can arise, that can escape the vaccine immune response, reducing efficacy, and the effectiveness of preventing onward transmission^18,19^. Effecting behaviour changes, that reduce the basic reproduction number, $\mathbb {R}$, of a population sufficiently, can be challenging^20^, as can countering the spread of misinformation about the benefits and risks of vaccination^21,22^. Because of this, there is a clear need to both calculate and articulate how these changes impact on an infected population.

Within the framework of a Susceptible-Infected-Recovered (SIR) model, that includes vaccination, the effectiveness of vaccination, and mortality, we have quantified the different benefits that vaccination provides at the direct level (of the individuals vaccinated) and at the indirect level (of the wider, unvaccinated community). We have analysed how vaccination produces *avoided deaths*, that is, deaths that *would* have occurred if vaccination had *not* been carried out. These measures allow exploration and understanding of how behaviour (under the proxy of the basic reproduction number), vaccination rate, and the vaccine’s effectiveness, jointly work together, to yield the benefits of vaccination. Through the production of this metric, we have shown that the benefits of vaccination go far beyond those attributed to the vaccinated individuals, and are sensitive to the value of $\mathbb {R}$, the vaccination rate and the vaccine’s effectiveness.

For a vaccine, we found that, regardless of its effectiveness, the direct benefit, $\Delta _{D}$ (see Eq. 5), increases linearly with the vaccination rate, *v*, to its maximum value at $\nu=1$. When multiplied by the mortality rate, *m*, the value of the maximum possible benefit (which occurs at $\nu=1$) turns out to be equal to the *attack rate* of the virus, namely the probability of death of an individual over the entire course of an epidemic^23^. When full vaccination ($\nu=1$) is achieved, all such deaths are avoided (assuming that vaccination reduces the risk of death, due to infection, to zero).

The indirect benefit of vaccination, $\Delta _{I}$ (see Eq. 6), on the other hand, has a different dependence on the vaccination rate, *v*. The indirect benefit, $\Delta _{I}$, first increases at low *v*, reaches a maximum, and then as *v* approaches 1 it decreases to zero. Interestingly, for the results presented above (where $\mathbb {R}=1.2$) the total benefits ($\Delta _{T}$, in terms of scaled avoided deaths) approaches the attack rate at relatively low *v*, on account of the large contribution of indirect benefits. A potentially important point needs to be made here about the population benefits of vaccination at these lower values of the vaccination rate, *v*, during a vaccination programme. The majority of the total benefit, in terms of avoided deaths, is attributable to the indirect effects at these low, initial values of *v* (cf. Fig. 4). However, we know from Fig. 2 that if $\mathbb {R}$ were to increase, the indirect benefit may be reduced. Consequently, any change in social behaviour, or policy during the early stages of a vaccination programme, which make it easier for a virus to transmit (e.g., easing social distancing rules, relaxation of mask-wearing), may reduce the indirect, population level benefit. However, even in the context of social restriction measures, which reduce the risk of infection in the wider, non-vaccinated population, there will be some level of indirect benefit arising from the effects of reduced transmission. It must be noted, however, that the effect of a varying $\mathbb {R}$, during the epidemic, has not been examined in this work.

Similarly, the effectiveness of a vaccine, $\varepsilon$, is a key determinant of how many deaths are avoided. As $\varepsilon$ decreases (i.e., mild infection and transmission is still possible in vaccinated individuals), as we saw in Fig. 3, the indirect benefits decrease, reducing the total benefits substantially at lower vaccination rates. Changing $\varepsilon$, did not however change the direct benefits. Again, this has important implications for policy, in that until vaccination rates are sufficiently high, and the direct benefits dominate, it would be potentially unwise to relax measures that reduce the likelihood of transmission.

## Supplementary Information

Supplementary Information.
