# Supplementary material for: Theoretically quantifying the direct and indirect benefits of vaccination against SARS-CoV-2 in terms of avoided deaths
Source: Sci Rep. 2022 May 25;12:8833. doi: 10.1038/s41598-022-12591-w (PMC9130994; doi:10.1038/s41598-022-12591-w)
Supplement: Supplementary file 1 — Supplementary Information. [file 41598_2022_12591_MOESM1_ESM.pdf]

SUPPLEMENTARY INFORMATION

Theoretically quantifying the direct and indirect benefits of vaccination against SARS-CoV-2 in Terms of Avoided Deaths

Greg Scutt[1], Michael Cross[2], and David Waxman[3]

[1] Medicines Optimisation Research Group, School of Applied Sciences, University of Brighton, Brighton, UK
[2]Pharmacy Department, Brighton and Sussex University Hospitals NHS Trust, Brighton, UK
[3]Centre for Computational Systems Biology, ISTBI, Fudan University, Shanghai, PRC

In this Supplementary Information we provide an exact formulation of the problem at hand, giving details of the dynamical model adopted. We then determine general expressions for the direct, indirect and total benefits of vaccination. The general expressions are supplemented with analytical approximations which expose their dependence on parameters of the model.

1 Exact formulation

We first provide an exact formulation of the problem, and in a later section provide approximate results.

1.1 Model

We work within the framework of a deterministic discrete time model of a population that is subject to a transmittable disease. The population consists of three categories of individuals labelled susceptible, infected and recovered. We incorporate effects of vaccination and mortality into the model, and this amounts to considering a variant of discrete time SIR dynamics (cf. [1]).

The following summarises the notation we use and incorporates key assumptions about the properties of the vaccine:

(i) n labels discrete time, and takes the values $0, 1, 2, ...$, with $n = 0$ the initial time;
(ii) S_n, I_n and R_n are the numbers, at time n, of individuals in the susceptible, infected and recovered categories, respectively;
(iii) N is the population size at time $n = 0$; we term this the *initial population size*;
(iv) $\mathbb{R}$ is the basic reproduction number, which is taken to be a constant (independent of time);
(v) p is the initial prevalence of the disease, i.e., the proportion of the population initially infected by the disease ($0 \leq p \leq 1$);
(vi) ν is the proportion of susceptible individuals who are vaccinated at the initial time, $n = 0$ ($0 \leq \nu \leq 1$);
(vii) ε is the effectiveness of the vaccine, i.e., the probability that a vaccinated individual is not susceptible to infection and transmission of the disease ($0 \leq \varepsilon \leq 1$); the probability that a vaccinated individual is susceptible to infection is $1 - \varepsilon$, and such individuals, as far as infection and disease transmission are concerned, are assumed indistinguishable from susceptible unvaccinated individuals;
(viii) m is the mortality rate of infected unvaccinated individuals, i.e., the probability an infected individual will die due to the disease ($0 \leq m \leq 1$); all vaccinated individuals are assumed to be protected from dying of the disease, irrespective of their susceptibility to infection, and hence have zero mortality due to the disease.

We begin with a population where, immediately before vaccination, there are $(1 - p)N$ susceptible individuals, pN infected individuals, and no recovered individuals. A proportion ν of the susceptible individuals are vaccinated at the initial time, $n = 0$. We shall next partition/reassign the number of individuals in the different categories, according to their susceptibility to infection. To this end, it is helpful to define

$$Q = (1 - p)N. \tag{1}$$

At time $n = 0$, immediately after vaccination, the following hold.

1. The number of unvaccinated individuals is $(1-\nu)Q$. These are all susceptible individuals.

2. The number of vaccinated individuals is νQ. This splits up into

$$\varepsilon\nu Q \text{ non-susceptible vaccinated individuals} \tag{2}$$

   and

$$(1-\varepsilon)\nu Q \text{ vaccinated individuals who remain susceptible.} \tag{3}$$

3. We reassign the $\varepsilon\nu Q$ non-susceptible vaccinated individuals directly to the recovered category, to reflect the role they play in the subsequent analysis.

4. The $(1-\varepsilon)\nu Q$ susceptible vaccinated individuals remain in the susceptible category.

5. The total number of individuals who are susceptible is the sum of: (i) the $(1-\nu)Q$ unvaccinated individuals (who are susceptible), and (ii) the $(1-\varepsilon)\nu Q$ vaccinated individuals who remain susceptible. Thus

$$\begin{aligned}\begin{array}{l}\text{total number of susceptible individuals,}\\ \text{immediately after vaccination}\end{array} &= (1-\nu)Q + (1-\varepsilon)\nu Q \\ &= (1-\varepsilon\nu)Q. \end{aligned} \tag{4}$$

6. The proportion of the initially susceptible individuals that are *not* vaccinated is written as f and given by

$$f = \frac{\text{number unvaccinated individuals}}{\text{total number of susceptible individuals}} = \frac{1-\nu}{1-\varepsilon\nu}. \tag{5}$$

We now proceed with establishing the dynamics.

We note that the pN individuals who were infected at time $n=0$ arose

purely from an unvaccinated population, and hence all of these individuals are subject to mortality at rate m.

Of the susceptible individuals present in the population, only a subset are subject to mortality, namely those who are unvaccinated; the vaccinated individuals who remain susceptible, despite the vaccine, are assumed to have zero mortality. Assuming individuals that become infected are picked at random from all susceptible individuals, it follows that the proportion of susceptible individuals that are unvaccinated *remains constant over time*, at the value f. As a consequence, of the individuals that ultimately become infected after time $n = 0$, a proportion f of these arise from unvaccinated individuals, and it is this fraction of infected individuals are subject to mortality from the disease at rate m.

The above considerations mean that the mortality rate at the initial time zero ($n = 0$) of infected individuals is simply m, but the mortality rate of infected individuals, for times greater than zero ($n > 0$), has to be multiplied by the factor f (the proportion of unvaccinated individuals) so that for infected individuals, the mortality has the effective value fm and we find it helpful to define

$$m_n = \begin{cases} m & \text{for } n = 0 \\ \\ fm & \text{for } n > 0. \end{cases} \tag{6}$$

The dynamics of the SIR model adopted here has the additional feature that each individual who is vaccinated will not die of the disease, but has a probability of permanently being susceptible to infection and transmission.

Susceptible individuals are those who have not been infected but can become infected in the future. Individuals that are infected at one particular time can transmit the disease, at this time, to susceptible individuals. At the next time these infected individuals have the possibilities of dying (if unvaccinated) or becoming recovered individuals, who can no longer can become infected. Infected individuals do not remain in the infected category after one time step.

The dynamics of the model then takes the form of the following system

of equations, which incorporates mortality and the effects of vaccination:

$$S_{n+1} = S_n - \frac{\mathbb{R} S_n I_n}{N} \tag{7}$$

$$I_{n+1} = \frac{\mathbb{R} S_n I_n}{N} \tag{8}$$

$$R_{n+1} = R_n + I_n - m_n I_n \tag{9}$$

where the initial numbers of individuals in the three categories are

$$S_0 = (1 - \varepsilon\nu)Q = (1 - \varepsilon\nu)(1 - p)N \tag{10}$$

$$I_0 = pN \tag{11}$$

$$R_0 = \varepsilon\nu Q = \varepsilon\nu(1 - p)N. \tag{12}$$

The number of individuals in the population at time n is $S_n + I_n + R_n$. When there is no mortality ($m = 0$), it follows from Eqs. (7), (8) and (9) that the number of individuals is constant (independent of n), but with mortality ($m \neq 0$) the number declines with n, and the total number of deaths that occur, over all time, is $\sum_{n=0}^{\infty} m_n I_n$.

1.2 Scaled dynamics

In what follows, we shall often work with the numbers of individuals in the three categories, when *scaled* by the initial population size, N. We define

$$s_n = \frac{S_n}{N} \tag{13}$$

$$i_n = \frac{I_n}{N} \tag{14}$$

$$r_n = \frac{R_n}{N}. \tag{15}$$

These obey the system of equations

$$s_{n+1} = s_n - \mathbb{R} s_n i_n \tag{16}$$

$$i_{n+1} = \mathbb{R} s_n i_n \tag{17}$$

$$r_{n+1} = r_n + i_n - m_n i_n \tag{18}$$

and take the initial values

$$s_0 = (1 - \varepsilon\nu)(1 - p) \tag{19}$$

$$i_0 = p \tag{20}$$

$$r_0 = \varepsilon\nu(1 - p). \tag{21}$$

Since the equations obeyed by the scaled variables s_n, i_n and r_n, along with their initial values, are all independent of the initial population size, N, it follows that s_n, i_n and r_n, all have the exact property of being independent of N for all times (all n).

It is appropriate to point out that in the wider context, the analysis we have presented is for a non-linear dynamical system, and a detailed analysis of related non-linear dynamical systems is given in [2].

1.3 Benefits of vaccination

There are direct and indirect benefits of vaccination. To determine these in a principled way, we compare two populations. One population is as described above, where vaccination has been carried out in a proportion ν of all susceptible individuals. In the other population, no vaccinations have been carried out, but in all other regards, the two populations are closely comparable (all parameter values except ν are identical).

1.3.1 Direct benefit

In the population where vaccination is carried out, the number of susceptible individuals that are vaccinated is νQ. The direct benefit of vaccination is measured by tracking the fate of the same number of individuals (namely νQ) that initially are in the susceptible category in an unvaccinated population. The number of deaths that ultimately occur to the νQ individuals in the unvaccinated population are deaths that do not occur in the corresponding vaccinated population, because, by assumption, vaccinated individuals are protected from dying of the disease. The deaths that occur amongst the νQ unvaccinated susceptible individuals are thus *avoided* by the vaccinated individuals, and are the *direct benefit* of vaccination.

To determine the direct benefit of vaccination, we first note that the fate of initially infected individuals is irrelevant to the direct benefit of vaccination, since these individuals are not among the susceptible individuals we are focussing upon.

Consider, then, the susceptible individuals in the unvaccinated population. The νQ susceptible individuals that we are tracking correspond, at time $n = 0$, to a proportion ν of all susceptible individuals. Assuming that infected individuals are drawn at random from susceptible individuals, it follows that *at any time*, n, those tracked individuals that remain in the sus-

ceptible category represent the same proportion, ν, of the total number of susceptible individuals at time n.

We apply this reasoning to the unvaccinated population. Of the individuals that were susceptible at time 0 and have become infected at time 1, a fraction ν of these arise from the individuals we are tracking. Similarly, of the individuals that were susceptible at time 1 and have become infected at time 2, a fraction ν of these arise from the individuals we are tracking. Continuing in this way, the total number of deaths that occur to the νQ individuals that we are tracking is $\nu m \sum_{n=1}^{\infty} I_n(0)$ where

$$I_n(0) \equiv I_n(\nu = 0) = \begin{array}{l} \text{No. of infected individuals in the} \\ \text{unvaccinated population at time } n. \end{array} \tag{22}$$

Thus vaccinating νQ susceptible individuals has the *benefit* of *directly* avoiding $\nu m \sum_{n=1}^{\infty} I_n(0)$ deaths. We can determine $I_n(0)$ from Eqs. (7) - (12) with the vaccination rate, ν, set to zero. It may be verified from Eq. (10), with ν set to zero, that $I_n(0)$ is independent of the effectiveness of the vaccine, ε, which is consistent with $I_n(0)$ being purely a property of an unvaccinated population, which knows nothing about properties of the vaccine.

A measure of the direct benefit of vaccination, that depends on a small set of parameters and contains core information about this quantity, is the number of directly avoided deaths, scaled by both the death rate, m, and the initial population size, N. We thus define

$$\Delta_D = \frac{\text{direct number of avoided deaths}}{mN} = \frac{\nu m \sum_{n=1}^{\infty} I_n(0)}{mN}$$

$$= \nu \sum_{n=1}^{\infty} i_n(0). \tag{23}$$

In what follows, we shall simply refer to Δ_D as the *direct benefit*. By Eqs. (16) - (21), Δ_D depends on the parameters $\mathbb{R}$, p and ν but not on N, m and ε, thus $\Delta_D \equiv \Delta_D(\mathbb{R}, p, \nu)$.

From Eq. (23), the ν dependence of $\Delta_D(\mathbb{R}, p, \nu)$ is particularly simple;

the $i_n(0)$ are properties of an unvaccinated population, and hence have no dependence on ν. Thus $\Delta_D(\mathbb{R}, p, \nu)$ is directly proportional to ν.

For an infected population, described by the parameters $\mathbb{R}$, p, N and m, vaccinating a proportion ν of susceptible individuals (i.e., vaccinating a total of $\nu(1-p)N$ such individuals) will have the direct effect of avoiding $Nm \times \Delta_D(\mathbb{R}, p, \nu)$ deaths.

1.3.2 Indirect benefit

In the population where vaccination is carried out, the number of susceptible individuals that are *not* vaccinated is $(1-\nu)Q$. The indirect benefit of vaccination is measured by tracking the fate of the same number of individuals (namely $(1-\nu)Q$) that initially are in the susceptible category in an unvaccinated population. The difference in numbers of deaths of the $(1-\nu)Q$ individuals in the unvaccinated and vaccinated populations represent deaths that are avoided, because of the *presence* of vaccinated individuals, and this difference constitutes the *indirect benefit* of vaccination.

We can use similar reasoning to that used when calculating the direct benefits of vaccination. The number of deaths among the $(1-\nu)Q$ initially susceptible individuals in the unvaccinated population is written $D(0) \equiv D(\nu = 0)$ and given by

$$D(0) = (1-\nu)m \sum_{n=1}^{\infty} I_n(0) \tag{24}$$

where $I_n(0)$ is given in Eq. (22).

The number of deaths of the $(1-\nu)Q$ unvaccinated individuals, in a population where vaccination has been carried out, requires a different argument.

We note that the fate of initially infected individuals is irrelevant to the indirect benefit of vaccination, since these individuals are not among the susceptible individuals that we are focussing upon.

Consider the vaccinated population at time $n = 0$ (immediately after vaccination). The susceptible individuals that are not vaccinated constitute a proportion f (Eq. (5)) of the total number of susceptible individuals. This

proportion of susceptible individuals, that are unvaccinated, remains constant over time at the value f, assuming individuals that become infected are picked at random from all susceptible individuals. As a consequence, of the individuals that ultimately become infected after time $n = 0$, a proportion f of these arise from unvaccinated individuals. These individuals have a probability of dying of m from the disease. The number of deaths of the initial number of $(1-\nu)Q$ unvaccinated susceptible individuals is written $D(\nu)$ and is thus given by

$$D(\nu) = fm \sum_{n=1}^{\infty} I_n(\nu) \tag{25}$$

where $I_n(\nu)$ is the number of infected individuals in the vaccinated population at time n. We can determine $I_n(\nu)$ from Eqs. (7) - (12) and note that there are two separate ways that ε enters $D(\nu)$: through the factor f in Eq. (25) and through the initial condition, Eq. (10).

There will be $D(0) - D(\nu) = (1-\nu)m \sum_{n=1}^{\infty} I_n(0) - fm \sum_{n=1}^{\infty} I_n(\nu)$ fewer (or avoided) deaths of unvaccinated individuals in a population where some individuals are vaccinated, compared with the deaths of corresponding individuals in an unvaccinated population. This reduction in the number of deaths, due to the presence of vaccinated individuals in the population, is the indirect benefit of vaccination.

Similar to the direct benefit of vaccination introduced above, we introduce a indirect benefit of vaccination, namely the number of indirectly avoided deaths, scaled by m and N. We thus define

$$\begin{aligned} \Delta_I &= \frac{\text{indirect number of avoided deaths}}{mN} = \frac{D(0) - D(\nu)}{mN} \\ &= \frac{(1-\nu)m \sum_{n=1}^{\infty} I_n(0) - fm \sum_{n=1}^{\infty} I_n(\nu)}{mN} \\ &= (1-\nu) \sum_{n=1}^{\infty} i_n(0) - f \sum_{n=1}^{\infty} i_n(\nu). \end{aligned} \tag{26}$$

In what follows, we shall refer to Δ_I simply as the *indirect benefit*. By Eqs. (16) - (21), Δ_I depends on the parameters $\mathbb{R}$, p, ε and ν, but not on N and m, thus $\Delta_I \equiv \Delta_I(\mathbb{R}, p, \varepsilon, \nu)$.

Consider an infected population described by the parameters $\mathbb{R}$, p, N and m. By vaccinating a proportion ν of susceptible individuals (i.e., vaccinating a total of $\nu(1-p)N$ such individuals) will have the indirect effect of avoiding $Nm \times \Delta_I(\mathbb{R}, p, \varepsilon, \nu)$ deaths.

1.3.3 Total benefit

The total benefit of vaccination is the sum of direct and indirect benefits. We write the total benefit, when scaled by m and N, as Δ_T. We have

$$\Delta_T = \text{total benefit } = \Delta_D + \Delta_I \tag{27}$$

$$= \nu \sum_{n=1}^{\infty} i_n(0) + (1-\nu) \sum_{n=1}^{\infty} i_n(0) - f \sum_{n=1}^{\infty} i_n(\nu)$$

$$= \sum_{n=1}^{\infty} i_n(0) - f \sum_{n=1}^{\infty} i_n(\nu). \tag{28}$$

Note that the difference between the total number of deaths that occur in an unvaccinated population, $m\sum_{n=0}^{\infty} I_n(0)$, and in a vaccinated population, $\sum_{n=0}^{\infty} m_n I_n(\nu)$, is proportional to Δ_T, the total benefit of vaccination. (The difference between the total number of deaths that occur in an unvaccinated population and a vaccinated population is $m\sum_{n=0}^{\infty} I_n(0) - \sum_{n=0}^{\infty} m_n I_n(\nu)$. This can be written as $(mpN + m\sum_{n=1}^{\infty} I_n(0)) - (mpN + \sum_{n=1}^{\infty} m_n I_n(\nu)) = m\sum_{n=1}^{\infty} I_n(0) - mf\sum_{n=1}^{\infty} I_n(\nu) = mN \times \Delta_T$).

2 Approximate results

We shall present some approximate results which give insight into the dependence of various quantities on parameters in the model and time.

2.1 Approximate form of the number of infected individuals

To obtain an approximation for the scaled number of infected individuals at time n, namely i_n, we begin by writing Eqs. (16) and (17) as

$$\begin{aligned} \ln(s_{n+1}) - \ln(s_n) &= \ln\left(1 - \mathbb{R}e^{\ln(i_n)}\right) \\ \ln(i_{n+1}) - \ln(i_n) &= \ln(\mathbb{R}) + \ln(s_n). \end{aligned} \tag{29}$$

We proceed by defining

$$A(n) = \ln(s_n) \tag{30}$$

$$B(n) = \ln(i_n) \tag{31}$$

and from Eq. (29), we have that

$$A(n+1) - A(n) = \ln\left(1 - \mathbb{R}e^{B(n)}\right) \tag{32}$$

$$B(n+1) - B(n) = \ln(\mathbb{R}) + A(n). \tag{33}$$

A simple approximate approach, which leads to solvable equations, is to: (i) assume $A(n)$ and $B(n)$ change slowly with n so they can be approximated as continuous functions of n; (ii) replace $A(n+1) - A(n)$ and $B(n+1) - B(n)$ by $dA(n)/dn$ and $dB(n)/dn$, respectively; (iii) approximate $\ln\left(1 - \mathbb{R}e^{B(n)}\right)$ by $-\mathbb{R}e^{B(n)}$. More sophisticated approximations of Eqs. (32) and (33) are possible, but we have not found another approximation that leads to simple analytic results. From the approximate approach, just described, we obtain

the equations

$$\frac{dA(n)}{dn} = -\mathbb{R}e^{B(n)} \tag{34}$$

$$\frac{dB(n)}{dn} = \ln(\mathbb{R}) + A(n). \tag{35}$$

Differentiating Eq. (35) with respect to n and using Eq. (34) yields the following self contained equation for $B(n)$:

$$\frac{d^2B(n)}{dn^2} + \mathbb{R}e^{B(n)} = 0. \tag{36}$$

The function $B(n)$ is subject to the initial conditions

$$B(0) = \ln(i_0) = \ln(p) \tag{37}$$

$$\left.\frac{dB(n)}{dn}\right|_{n=0} = \ln(\mathbb{R}s_0) = L \tag{38}$$

where we have defined

$$L = \ln\left[\mathbb{R}(1-\varepsilon\nu)(1-p)\right]. \tag{39}$$

To solve Eq. (36) we multiply by $2\frac{dB(n)}{dn}$, and obtain $\frac{d}{dn}\left[\left(\frac{dB}{dn}\right)^2 + 2\mathbb{R}e^B\right] = 0$. Thus $\left(\frac{dB}{dn}\right)^2 + 2\mathbb{R}e^B$ takes an n independent (i.e., constant) value that we write as α^2, since it is positive. We thus have $\left(\frac{dB}{dn}\right)^2 + 2\mathbb{R}e^B = \alpha^2$. This leads to the equation $\frac{dB}{dn} = \alpha\sqrt{1 - \frac{2\mathbb{R}}{\alpha^2}e^B}$ whose solution is

$$B(n) = \ln\left[\frac{\alpha^2}{2\mathbb{R}}\operatorname{sech}^2\left(\frac{\alpha}{2}n + \frac{\beta}{2}\right)\right] \tag{40}$$

where β (like α) is an arbitrary constant. Without loss of generality we can take $\alpha > 0$, while β may take negative or positive values.

The values of α and β are determined from the initial conditions given in Eqs. (37) and (38), which lead to $\operatorname{sech}^2\left(\frac{\beta}{2}\right) = \frac{2\mathbb{R}p}{\alpha^2}$ and $\tanh\left(\frac{\beta}{2}\right) = -\frac{L}{\alpha}$,

respectively. Rewriting these equations using $\text{sech}^2 = 1 - \tanh^2$, yields

$$\alpha = \sqrt{2\mathbb{R}p + L^2} \tag{41}$$

$$\tanh\left(\frac{\beta}{2}\right) = -\frac{L}{\sqrt{2\mathbb{R}p + L^2}} \tag{42}$$

with this last result is equivalent to

$$\beta = \ln\left(\frac{\sqrt{2\mathbb{R}p + L^2} - L}{\sqrt{2\mathbb{R}p + L^2} + L}\right). \tag{43}$$

Using Eqs. (31) and (40) it follows that the scaled number of infections is approximately given by

$$i_n = \frac{\alpha^2}{2\mathbb{R}} \text{sech}^2\left(\frac{\alpha}{2}n + \frac{\beta}{2}\right). \tag{44}$$

We note that the behaviour of i_n is sensitive to the sign of β, and by Eq. (42) the sign of β is opposite the sign of L. If $\beta > 0$ (equivalently, $L < 0$), then i_n is a decreasing function of n. If $\beta < 0$ (equivalently, $L > 0$) then i_n first increases with n, achieves a maximum, and then decreases with n. This is the case shown in Figure 1 of the main text, and is qualitatively similar to the behaviour seen in continuous time SIR models, for the number of infections over time, when the number of infections starts at a low value. Note that for large n, both signs of β lead to i_n approaching zero as $i_n \sim \frac{2\alpha^2 e^{-\beta}}{\mathbb{R}} e^{-\alpha n}$.

2.1.1 Scaled benefits

The direct benefit of vaccination is given by Eq. (23). Using the approximate form of i_n given in Eq. (44) we can derive an approximation for the direct

benefit, $\Delta_D(\mathbb{R}, p)$. We have

$$\Delta_D(\mathbb{R}, p, \nu) = \nu \sum_{n=1}^{\infty} i_n(0) \simeq \nu \int_0^{\infty} \frac{\alpha_0^2}{2\mathbb{R}} \operatorname{sech}^2\left(\frac{\alpha_0}{2} n + \frac{\beta_0}{2}\right) dn$$

$$= \nu \frac{\alpha_0}{\mathbb{R}} \left[1 - \tanh\left(\frac{\beta_0}{2}\right)\right]$$

$$= \nu \frac{\alpha_0 + L_0}{\mathbb{R}} \tag{45}$$

where we have made use of Eq. (42) and defined

$$L_0 = \ln\left[\mathbb{R}(1-p)\right] \text{ and } \alpha_0 = \sqrt{2\mathbb{R}p + L_0^2}. \tag{46}$$

The indirect benefit of vaccination is given by Eq. (26) and we again use the approximate form of i_n given in Eq. (44). Proceeding as in the case of the direct benefit we obtain

$$\Delta_I(\mathbb{R}, p, \varepsilon, \nu) = (1-\nu) \sum_{n=1}^{\infty} i_n(0) - f \sum_{n=1}^{\infty} i_n(\nu)$$

$$\simeq (1-\nu) \frac{\alpha_0 + L_0}{\mathbb{R}} - f \frac{\alpha + L}{\mathbb{R}}. \tag{47}$$

Lastly, the total benefit is given by $\Delta_T = \Delta_D + \Delta_I$ i.e.,

$$\Delta_T(\mathbb{R}, p, \varepsilon, \nu) \simeq \frac{\alpha_0 + L_0}{\mathbb{R}} - f \frac{\alpha + L}{\mathbb{R}}. \tag{48}$$

The dependence of Δ_D on ν is linear for all ν, but for comparison we give

it here, along with small ν ($\nu \ll 1$) results for Δ_I and Δ_T:

$$\Delta_D \simeq \frac{\nu}{\mathbb{R}} \left(\alpha_0 + L_0\right) \tag{49}$$

$$\Delta_I \simeq \frac{\nu}{\mathbb{R}} \varepsilon \left(\alpha_0 + L_0\right) \frac{(1-\alpha_0)}{\alpha_0} + O(\nu^2) \tag{50}$$

$$\Delta_T \simeq \frac{\nu}{\mathbb{R}} \left(\alpha_0 + L_0\right) \left(1 + \varepsilon \frac{1-\alpha_0}{\alpha_0}\right) + O(\nu^2). \tag{51}$$

For small ν, the range of $\mathbb{R}$ and ε where Δ_I exceeds Δ_D is given by $\Delta_I/\Delta_D \geq 1$, which can be written as

$$\varepsilon \geq \frac{\alpha_0}{1-\alpha_0} \text{ or } \alpha_0 \leq \frac{\varepsilon}{1+\varepsilon}. \tag{52}$$

As ν approaches 1 we note that Δ_D and Δ_T approach the same constant $\left(\alpha_0 + L_0\right)/\mathbb{R}$, but Δ_I vanishes as $(1-\nu)$.

Reference

[1] Allen L. J. (1994)
Some discrete-time si, sir, and sis epidemic models
Mathematical Biosciences 124(1):83-105.

[2] Wang W, Cai Y, Wu M, Wang K, Li Z. (2012)
Complex dynamics of a reaction–diffusion epidemic model.
Nonlinear Analysis: Real World Applications 13:2240–2258.
